# Effective automatic classification methods via deep learning for myopic maculopathy

**DOI:** 10.3389/fmed.2024.1492808

**Published:** 2024-11-13

**Authors:** Zheming Zhang, Qi Gao, Dong Fang, Alfira Mijit, Lu Chen, Wangting Li, Yantao Wei

**Affiliations:** ^1^State Key Laboratory of Ophthalmology, Zhongshan Ophthalmic Center, Sun Yat-sen University, Guangzhou, China; ^2^School of Future Technology, South China University of Technology, Guangzhou, China; ^3^Pazhou Lab, Guangzhou, China; ^4^Shenzhen Eye Hospital, Jinan University, Shenzhen, China

**Keywords:** myopic maculapathy, ensemble learning, deep learning, artificial intelligence, fundus image

## Abstract

**Background:**

Pathologic myopia (PM) associated with myopic maculopathy (MM) is a significant cause of visual impairment, especially in East Asia, where its prevalence has surged. Early detection and accurate classification of myopia-related fundus lesions are critical for managing PM. Traditional clinical analysis of fundus images is time-consuming and dependent on specialist expertise, driving the need for automated, accurate diagnostic tools.

**Methods:**

This study developed a deep learning-based system for classifying five types of MM using color fundus photographs. Five architectures—ResNet50, EfficientNet-B0, Vision Transformer (ViT), Contrastive Language-Image Pre-Training (CLIP), and RETFound—were utilized. An ensemble learning approach with weighted voting was employed to enhance model performance. The models were trained on a dataset of 2,159 annotated images from Shenzhen Eye Hospital, with performance evaluated using accuracy, sensitivity, specificity, F1-Score, Cohen’s Kappa, and area under the receiver operating characteristic curve (AUC).

**Results:**

The ensemble model achieved superior performance across all metrics, with an accuracy of 95.4% (95% CI: 93.0–97.0%), sensitivity of 95.4% (95% CI: 86.8–97.5%), specificity of 98.9% (95% CI: 97.1–99.5%), F1-Score of 95.3% (95% CI: 93.2–97.2%), Kappa value of 0.976 (95% CI: 0.957–0.989), and AUC of 0.995 (95% CI: 0.992–0.998). The voting ensemble method demonstrated robustness and high generalization ability in classifying complex lesions, outperforming individual models.

**Conclusion:**

The ensemble deep learning system significantly enhances the accuracy and reliability of MM classification. This system holds potential for assisting ophthalmologists in early detection and precise diagnosis, thereby improving patient outcomes. Future work could focus on expanding the dataset, incorporating image quality assessment, and optimizing the ensemble algorithm for better efficiency and broader applicability.

## 1 Introduction

Pathologic myopia (PM) is one of the leading causes of visual impairment and blindness worldwide (1, 2). Over the past half-century, the prevalence of myopia has increased significantly, particularly in East Asia, where the proportion of high myopia cases has also risen. In these regions, up to 80% of 18-year-old high school graduates are myopic, with 20% of these cases classified as high myopia (3). The higher the degree of myopia, the greater the risk of developing PM. The growing incidence of PM, along with its associated severe ocular complications, underscores the critical need for effective screening and management strategies in global public health.

According to the meta-analysis for pathologic myopia (META-PM) classification system proposed by Ohno-Matsui et al., PM is defined as the presence of severe ocular lesions in fundus photographs that are equivalent to or exceed diffuse chorioretinal atrophy, or features such as lacquer cracks, myopic choroidal neovascularization (CNV), and Fuchs’ spots (4). Due to the irreversible pathological changes in the shape and structure of the myopic eye, effective treatment options for PM remain limited, and the prognosis for PM-related complications is generally poor. Additionally, the slow progression of PM often leads patients to overlook symptoms, attributing them instead to issues with their corrective lenses, thus delaying diagnosis (5). Early diagnosis allows timely intervention and follow-up screenings, helping patients understand their condition and take a proactive role in managing their health. This is key to preventing further deterioration and improving outcomes. Therefore, regular screening of myopic individuals to detect PM early and prevent its progression is of paramount importance.

Fundus imaging has become a vital tool in ophthalmic diagnostics for common eye diseases due to its non-invasive, accessible, and easily processed nature (6). However, traditional clinical image analysis heavily relies on doctors’ expertise and experience and is time-consuming (7). This has driven the development of efficient, automated, and accurate fundus image analysis systems, which are critical strategies for the future of preventing and treating eye diseases.

In recent years, artificial intelligence (AI) and deep learning technologies have advanced rapidly in the field of medical image processing (8–10), leading to the emergence of new techniques for analyzing fundus images related to high myopia (11, 12). AI can utilize structural changes in the eye, particularly those linked to high myopia, to predict specific conditions. High myopia is typically associated with the elongation of the eyeball and alterations in the retina, which can lead to various retinal complications. The correlation between ocular structure and conditions like high myopia highlights the significance of analyzing fundus images for predictive diagnostics. For instance, a recent study demonstrated that fundus photography can estimate corneal curvature, a crucial factor in refractive errors, showcasing AI’s ability to extract valuable insights from these images (13). By recognizing these structural variations, AI models can greatly enhance their predictive accuracy and improve patient outcomes in myopic disease contexts.

These technologies hold significant potential in assisting ophthalmologists by enhancing diagnostic efficiency and accuracy. For instance, Cen et al. developed a deep learning platform capable of detecting 39 different fundus diseases and conditions, demonstrating excellent performance in multi-label classification tasks (14). Similarly, Li et al. proposed the MyopiaDTER model, which introduced a novel attention feature pyramid networks (FPN) architecture and generated multi-scale feature maps for the traditional detection transformer (DETR), enabling the detection of normal myopia, high myopia, and pathologic myopia regions in fundus photographs, achieving three-class classification (15).

Moreover, due to the often limited availability of medical data, self-supervised learning is expected to gain significant traction in the field (16). In this regard, Zhou et al. introduced the RETFound model, trained on 1.6 million unlabeled retinal images using self-supervised learning and later adapted for disease monitoring tasks with labeled data (17). The model demonstrated superior performance compared to several baselines in diagnosing and predicting sight-threatening eye diseases, establishing itself as a foundational tool for ophthalmic image analysis.

Ensemble learning has demonstrated significant potential in various medical applications. For instance, Namamula and Chaytor proposed an ensemble learning approach that combined the results of the Edge Detection Instance Preference (EDIP) algorithm with Extreme Gradient Boosting (XGBoost), leading to enhanced accuracy in analyzing large-scale medical datasets. Their method achieved impressive success rates in diagnosing conditions such as blood cancer and diabetes (18).

In this study, we explore an effective automatic recognition system for pathologic myopia-related fundus lesions. We utilize five deep learning architectures to train models capable of recognizing five types of myopic maculopathy (MM) using color fundus photographs. The five architectures include ResNet50 (19) and EfficientNet-B0 (20), both of which have been proven effective in medical classification tasks; Vision Transformer (ViT) (21), which utilizes advanced transformer units for image feature extraction and analysis; Contrastive Language-Image Pre-Training (CLIP) (22) model, which enhances image understanding through language-vision alignment; and RETFound (17), which has been pre-trained on a large dataset of fundus images.

To enhance the system’s accuracy and reliability, we employ an ensemble learning approach, integrating the outputs of these models through a weighted voting strategy (23). This research not only aims to reduce the workload of clinicians and address the shortage of medical resources but also enables rapid screening of pathologic myopia-related fundus lesions, serving a wide population and providing significant clinical and social value.

## 2 Materials and methods

### 2.1 Data

This study collects a total of 2,159 original color retinal fundus images from Shenzhen Eye Hospital, with analysis commencing in June 2024. The images are captured using a desktop non-mydriatic retinal camera. All images are with a field of view 45 degrees, centered on the macula or on the connecting center of optic disc and macula. There are no quality issues in the images collected, such as images with obscured macular areas due to severe artifacts, defocus blurring or inadequate lighting, and with incorrect field position.

**Figure 1:**
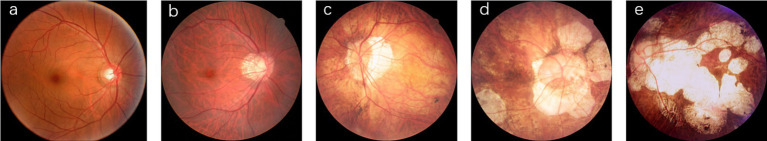
Representative images of five myopic macular degeneration categories. (a) no myopic retinopathy, (b) tessellated fundus, (c) diffuse chorioretinal atrophy, (d) patchy chorioretinal atrophy, (e) macular atrophy.

According to the META-PM classification system (4), MM is categorized into five grades (shown in Figure 1): no myopic retinopathy (C0), tessellated fundus (C1), diffuse chorioretinal atrophy (C2), patchy chorioretinal atrophy (C3), and macular atrophy (C4). Additionally, lacquer cracks, CNV, and Fuchs’ spots are defined as “Plus” lesions. Grade C1 is characterized by distinct choroidal vessels visible around the fovea and arcade vessels. Grade C2 presents with a yellowish-white appearance of the posterior pole, with atrophy assessed relative to the optic disc area. Grade C3 is marked by well-defined gray-white lesions in the macular region or around the optic disc. Grade C4 features well-defined, gray-white or white, round atrophic lesions in the foveal region. Grades C0 and C1 indicate low-risk high myopia, while Grades C2-C4 represent high-risk high myopia, also known as PM. In PM fundus images, “Plus” lesion features may be observed, which are not specific to any particular grade but can develop from or occur within any grade. This study primarily focuses on the five-class classification task among Grades C0-C4.

The color fundus photographs are annotated by two professional ophthalmologists according to the aforementioned classification system, with the distribution of the dataset detailed in Table 1.

**Table 1:** Distribution of the dataset.

Category	Number
C0	510
C1	678
C2	401
C3	408
C4	162
Total	2,159

### 2.2 Image preprocessing

To minimize the interference of black regions in fundus images on feature extraction, redundant black areas in the images are cropped. First, the images are loaded using the OpenCV library and converted to grayscale as follows:


$$I_{gray}=0.299\times I_{R}\left(x,y\right)+0.587\times I_{G}\left(x,y\right)+0.114\times I_{B}\left(x,y\right)$$

where $I_{R}\left(x,y\right)$ , $I_{G}\left(x,y\right)$ , and $I_{B}\left(x,y\right)$ respectively represent the value of pixel (x,y) in the red, green and blue channels. Subsequently, a binary mask M(x,y) is generated using thresholding:


$$M\left(x,y\right)=\{\begin{array}{c} 255\;\;I_{gray}\left(x,y\right)\geq 20\\ 0\;otherwise \end{array}$$

By detecting the largest contour in the mask, the coordinates of its minimum bounding box $\left(x_{m},y_{m},w,h\right)$ are calculated, and the region within this bounding box is cropped:


$$ROI=I\left[y_{m}:y_{m}+h,x_{m}:x_{m}+w\right]$$

This process results in a fundus image with black regions removed, preserving the relevant retinal information. Prior to developing the deep learning system, image normalization is performed. All fundus images are normalized to pixel values within the range of 0–1 and resized to a resolution of 224 × 224 pixels.

### 2.3 Development of the deep learning system

During the system development, the test set is constructed using a stratified random sampling method, where 20% of data from each category is randomly selected to form the test set. The remaining data is used for model training and validation through five-fold cross-validation. Specifically, the remaining data is randomly divided into five equally sized folds, ensuring that each image appeared in only one fold. The training process is conducted in two steps: first, four folds are selected for algorithm training and hyperparameter optimization, while the remaining fold is used for validation. This process is repeated five times, ensuring that each fold served as a validation set. This approach aims to ensure balanced data distribution across folds and effectively evaluate the model’s generalization ability.

The model training involves five different architectures: ResNet50 (19), EfficientNet-B0 (20), ViT (24), CLIP (22), and RETFound (17). These architectures are chosen due to their superior performance in visual tasks and their success in similar research. Specifically, ResNet50 and EfficientNet-B0 excel in feature extraction and computational efficiency, Vision Transformer is effective in handling global image information, CLIP performs well in image-text matching and image understanding, and RETFound demonstrates outstanding performance in retinal image analysis. To initialize weights and leverage existing knowledge, the first four models are pre-trained on the ImageNet Large Scale Visual Recognition Challenge (25), a comprehensive database with 1.28 million images classified into 1,000 categories. The RETFound model is pre-trained through self-supervised learning on 1.6 million retinal images and validated across various disease detection tasks.

For the CLIP model, fine-tuning is performed by aligning image and text features through contrastive learning. Class labels are used as textual descriptions during training, enabling the model to adapt to our specific task even with limited data. Similarly, other models are fine-tuned by initializing from pretrained weights and applying transfer learning, with five-fold cross-validation used to adapt them to our classification problem.

Our training platform utilize the PyTorch framework, with all deep learning algorithms run on a NVIDIA 4090 graphics processing unit (GPU) (26). The batch size is set to 32, and model parameters are updated based on the mean of the samples. The training process employ the AdamW (27) optimizer with weight decay, with a learning rate set at 0.001. Each model is trained for 50 epochs, and performance is monitored at the end of each epoch using metrics including loss, accuracy, sensitivity, specificity, F1-Score, Kappa, and AUC on the validation dataset. The best model parameters that show the highest AUC on the validation set are saved.

Finally, we employ an ensemble learning approach using the voting strategy to combine the predictions of ResNet50 (19), EfficientNet-B0 (20), ViT (24), CLIP (22), and RETFound (17). The predictions are combined through weighted voting, where each model’s voting weight is adjusted according to its average AUC score on the five-fold validation sets. The final classification result is determined by the ensembled prediction probability $prob_{final}$ .

$$w_{m}=\frac{1}{5}\overset{5}{\underset{v=1}{\sum }}AUC_{m,v}\;\;\;m\in \left\{\mathrm{ResNet}50\mathrm{,EfficientNet}-B0\mathrm{,ViT,CLIP,RETFound}\right\}$$

$$prob_{final}=\underset{m}{\sum }\overset{5}{\underset{v}{\sum }}w_{m}\cdot prob_{m,v}$$

where $w_{m}$ represents the weight of the method *m*. v denotes the *v*th fold. $AUC_{m,v}$ represents the AUC on the verification set for the model under the *v*th fold among the method *m*. $prob_{m,v}$ is the probability of the model under the *v*th fold among the method *m.* This method is expected to enhance the overall accuracy and robustness of the system and improve the model’s generalization ability on the test set.

### 2.4 Evaluation of the AI system

To comprehensively evaluate the classification performance of the models, this study employs a range of metrics including accuracy, sensitivity, specificity, F1-Score, weighted Cohen’s Kappa, and AUC, with 95% confidence intervals (CI) calculated for all metrics. All metrics are derived from the results of five-fold cross-validation, and the average values are computed across the folds.

In multi-category classification task, sensitivity and specificity are calculated using a one-*vs*-rest strategy. The 95% CI for accuracy, sensitivity, and specificity are estimated using the Wilson Score method implemented in the Statsmodels package (version 0.13.5). For the F1-score, weighted Cohen’s Kappa, and AUC, the 95% CI are calculated using the empirical Bootstrap method (28), with 1,000 resamples performed to ensure the robustness of the results.

In addition to numerical metrics, model performance is assessed through visualization techniques. The receiver operating characteristic (ROC) curve illustrates the model’s performance across different threshold values, with an AUC value closer to 1.0 indicating better classification capability. The confusion matrix compares the true labels with the predicted labels, clearly displaying the number of correct and incorrect classifications for each category. The ROC curves and confusion matrices are plotted using Matplotlib (version 3.8.3) and Scikit-learn (version 1.4.1) libraries.

### 2.5 Interpretability of AI system

To better understand the impact of different regions of fundus images on classification results, identify the causes of misclassification, and enhance the interpretability of the model, we employ visualization techniques to analyze the convolutional network model used in our experiments. Class Activation Mapping (CAM) (29) is a visualization technique that aggregates feature maps weighted by network parameters to generate heatmaps, which highlight the importance of each pixel in the image classification process. In these heatmaps, more important regions are indicated with warmer colors. However, CAM requires modifications to the network architecture and retraining of the model. To simplify implementation, this study utilizes Grad-CAM++ (30), which does not require any changes to the network structure. Grad-CAM++ provides a clear visualization of the features learned by the model while maintaining classification accuracy, making the model more transparent and interpretable.

## 3 Results

### 3.1 Evaluation of deep learning models

This study evaluates the performance of five deep learning models (ResNet50, EfficientNet-B0, ViT, CLIP, and RETFound) and their ensemble results for classifying five types of MM. All models are trained and validated using five-fold cross-validation and assessed on an independent test dataset. Evaluation metrics include accuracy, sensitivity, specificity, F1-Score, weighted Cohen’s Kappa, and AUC, all reported with 95% CI. The models are then combined using a weighted voting ensemble approach. Table 2 presents the performance of each model as well as the results of the weighted voting ensemble.

**Table 2:** Performance of individual models and their voting ensemble for classifying myopic maculopathy.

Model	Accuracy (95% CI)	Sensitivity (95% CI)	Specificity (95% CI)	F1-Score (95% CI)	Kappa (95% CI)	AUC (95% CI)
ResNet50	91.8%	91.8%	97.9%	91.7%	0.958	0.991
	(90.5, 92.8%)	(87.4, 93.0%)	(97.2, 98.5%)	(90.5, 92.8%)	(0.948, 0.966)	(0.988, 0.993)
EfficientNet-B0	91.2%	91.2%	97.8%	91.0%	0.964	0.990
	(89.9, 92.3%)	(86.4, 92.0%)	(97.0, 98.4%)	(89.8, 92.3%)	(0.956, 0.970)	(0.987, 0.992)
ViT	92.4%	92.4%	98.1%	92.3%	0.960	0.986
	(91.2, 93.5%)	(86.9, 92.7%)	(97.3, 98.6%)	(91.2, 93.5%)	(0.950, 0.968)	(0.982, 0.989)
CLIP	91.7%	91.7%	97.9%	91.6%	0.961	0.989
	(90.5, 92.8%)	(85.4, 91.4%)	(97.2, 98.5%)	(90.3, 92.8%)	(0.952, 0.968)	(0.987, 0.992)
RETFound	91.8%	91.8%	97.9%	91.6%	0.962	0.990
	(90.5, 92.8%)	(85.6, 91.4%)	(97.2, 98.5%)	(90.4, 92.7%)	(0.954, 0.970)	(0.987, 0.992)
Voting	**95.4%**	**95.4%**	**98.9%**	**95.3%**	**0.976**	**0.995**
	**(93.0, 97.0%)**	**(86.8, 97.5%)**	**(97.1, 99.5%)**	**(93.2, 97.2%)**	**(0.957, 0.989)**	**(0.992, 0.998)**

Bold indicates the best result.

The voting ensemble algorithm shows the best performance across all evaluation metrics. Specifically, the voting algorithm achieves an accuracy of 95.4% (95% CI: 93.0–97.0%), sensitivity of 95.4% (95% CI: 86.8–97.5%), specificity of 98.9% (95% CI: 97.1–99.5%), F1-Score of 95.3% (95% CI: 93.2–97.2%), Kappa value of 0.976 (95% CI: 0.957–0.989), and AUC of 0.995 (95% CI: 0.992–0.998).

**Figure 2:**
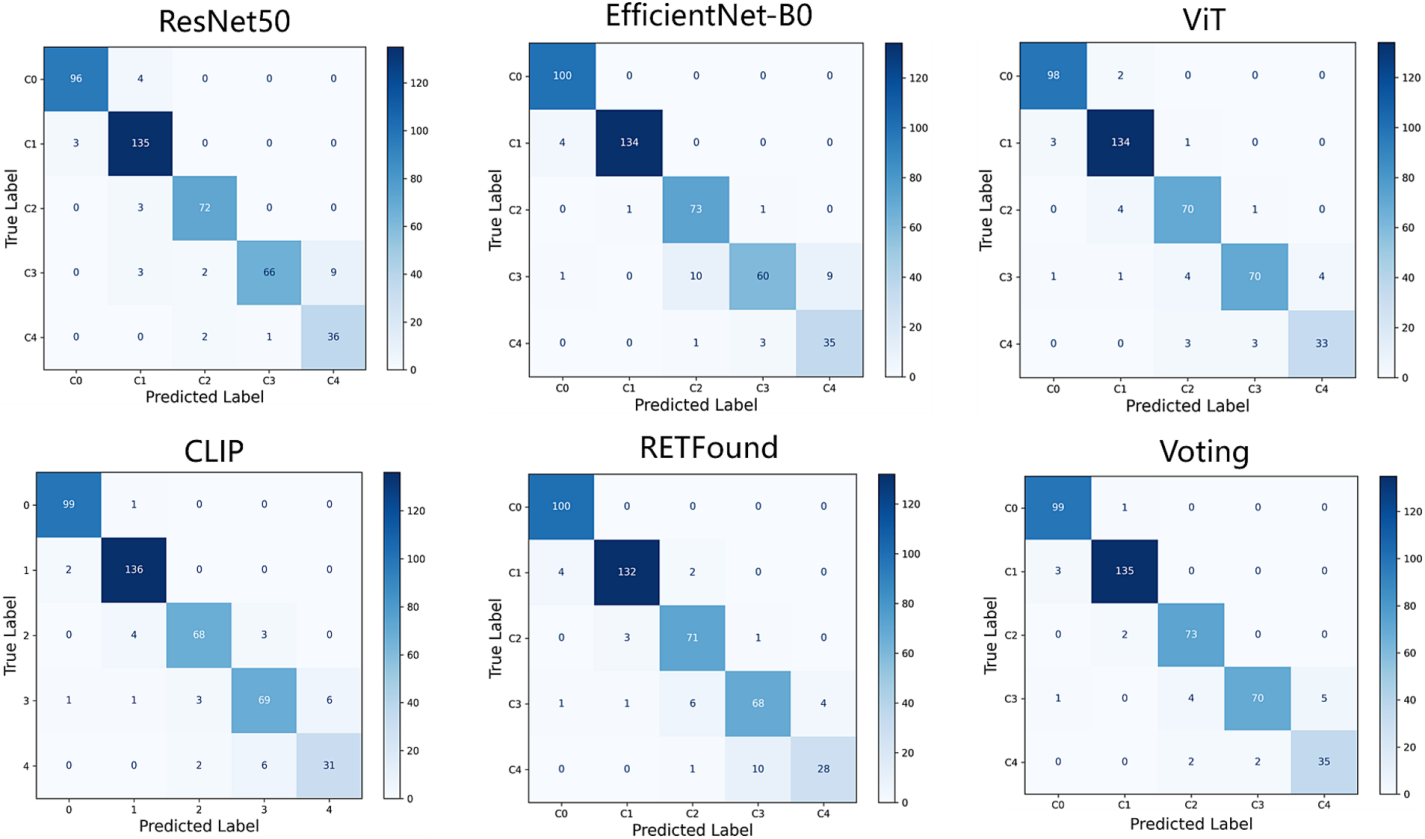
Confusion matrices for five models and their voting ensemble.

**Figure 3:**
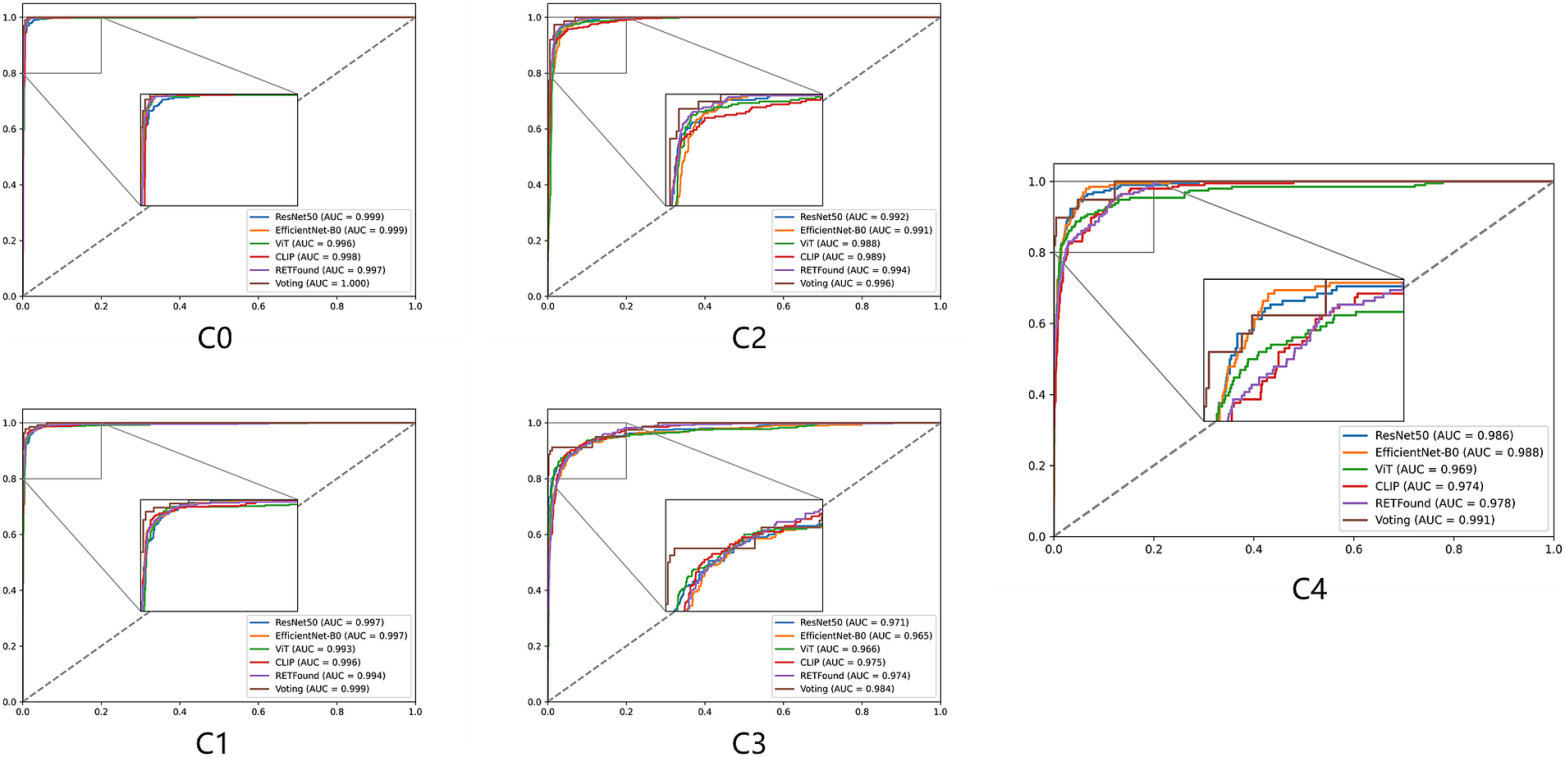
ROC curves for five models and their voting ensemble across five myopic maculopathy categories. C0 no myopic retinal pathology, C1 tessellated fundus, C2 diffuse chorioretinal atrophy, C3 patchy chorioretinal atrophy, and C4 macular atrophy.

Figures 2, 3 display the performance of the five deep learning models and their voting ensemble results on the test set. From the confusion matrix (Figure 2), EfficientNet-B0 and RETFound models achieve the highest classification accuracy in the C0 class; the CLIP model performs best in the C1 class; EfficientNet-B0 model and voting strategy excel in the C2 class; ViT model and voting strategy are the best in the C3 class; and ResNet50 performs best in the C4 class. Although the voting strategy does not show the highest accuracy in every category, its accuracy is consistently high across categories, demonstrating strong robustness. Furthermore, from the ROC curves for each category (Figure 3), the voting ensemble method also performs better, with the highest AUC values for all categories, further confirming its effectiveness in complex lesion classification tasks. Additionally, it can be observed that most of the misclassified images were assigned to adjacent categories, which were largely due to the visual similarity between categories, making it difficult for the model to distinguish between them.

The inference time of the ensemble model is the sum of the inference times of the individual models, plus the time required for the ensemble process following each model’s prediction. Table 3 provides detailed timing for each fold of every model when processing individual images. The ensemble process itself takes approximately 5.7 ms, demonstrating that the computational cost and time required for weighted voting in the ensemble are minimal.

**Table 3:** Inference times for individual models (per image).

Model	Inference time per image (ms)
ResNet50	46.2
EfficientNet-B0	33.1
ViT	40.6
CLIP	49.6
RETFound	97.8

t-SNE (34) is a commonly used technique for dimensionality reduction and visualization of high-dimensional data, helping us to visually observe the distribution of different categories in the feature space. Figure 4 shows the t-SNE plots for each model, which reveal that the scatter points for each category are relatively concentrated, with some overlap between adjacent categories. For example, the red points (C4) contain many yellow points (C3), indicating that the C3 class is prone to being misclassified as C4. However, the t-SNE plot for the CLIP model shows that points of the same category also appear in multiple clusters. Particularly, the boundary between red (C4) and yellow (C3) points is not very clear, suggesting that distinguishing between these two categories is challenging. This overlap suggests that the visual similarity between these categories affects the model’s performance.

**Figure 4:**
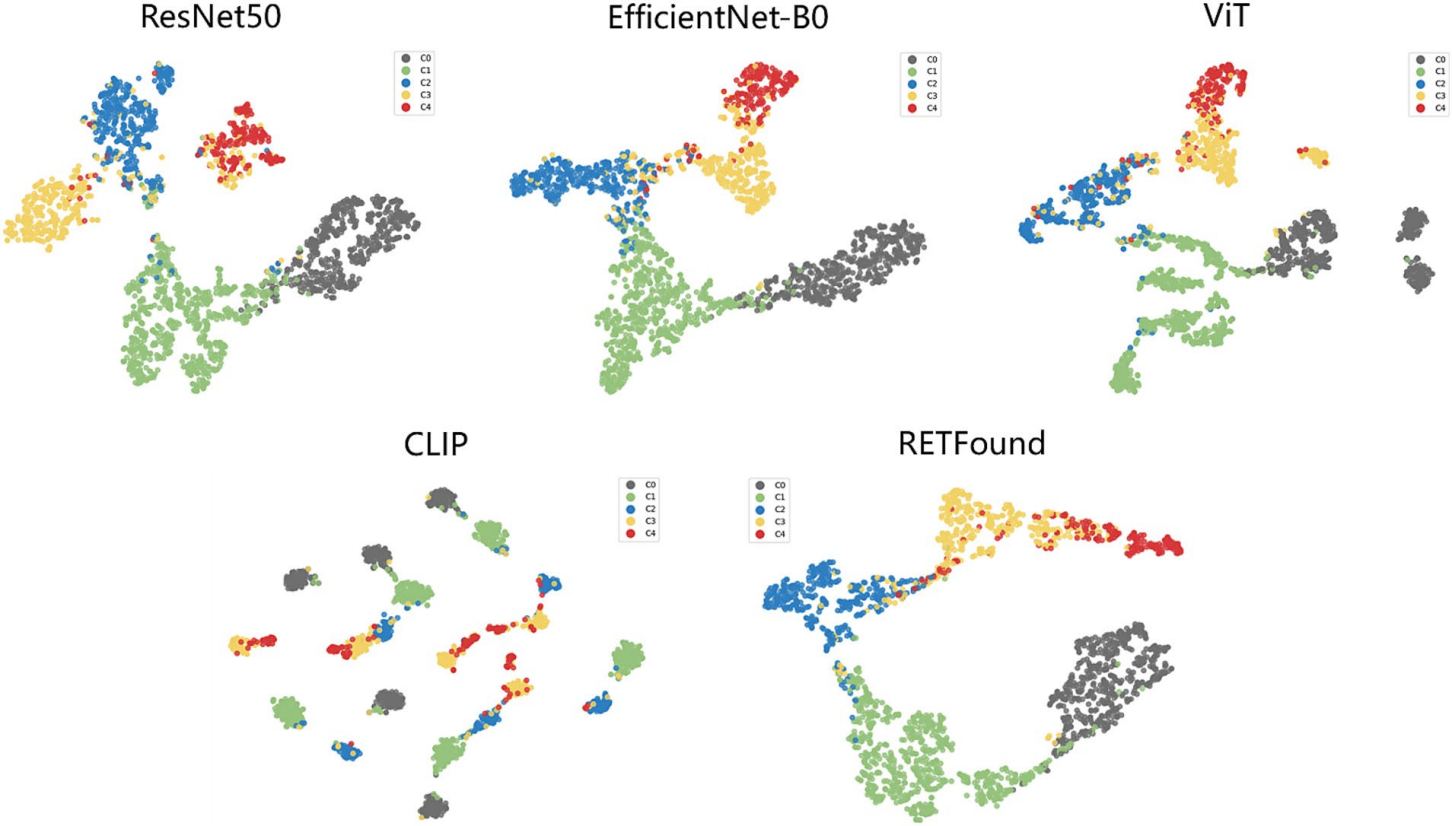
t-SNE visualization of embedding features for five models. The embedding features learned by the deep learning models from the test dataset are projected into 2D using t-SNE, with points representing category distributions and different colors indicating different categories.

Additionally, the dataset predominantly contains images with single lesions, which may limit the model’s ability to generalize to cases with coexisting multiple lesions. Expanding the dataset to include such cases would improve model robustness and applicability in real-world clinical scenarios.

### 3.2 Classification errors

**Figure 5:**
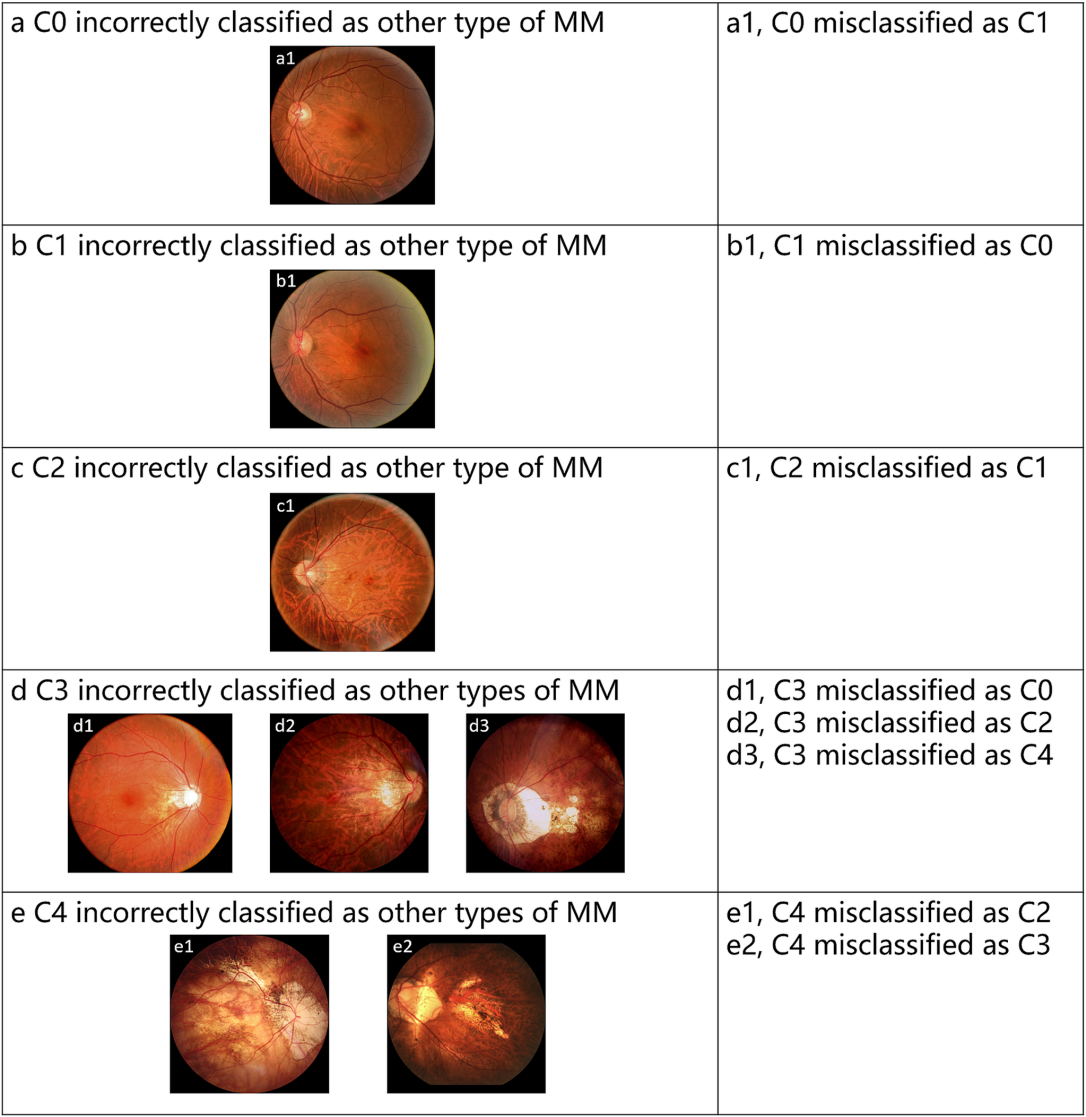
Representative examples of misclassified images by voting ensemble. (a–e) Represent the misclassified images of C0-C4, respectively.

The test set contains 432 images, with 20 images (4.63% of the total) showing inconsistencies between the Voting ensemble results and the reference standards. Specifically, the voting ensemble method misclassified 1 image from class C0, 3 images from class C1, 2 images from class C2, 10 images from class C3, and 4 images from class C4. These errors were mainly due to the visual similarity of features between certain classes, making it challenging for the model to distinguish them. Figure 5 provides examples of typical images incorrectly classified by the voting ensemble.

### 3.3 Visual interpretation of models

**Figure 6:**
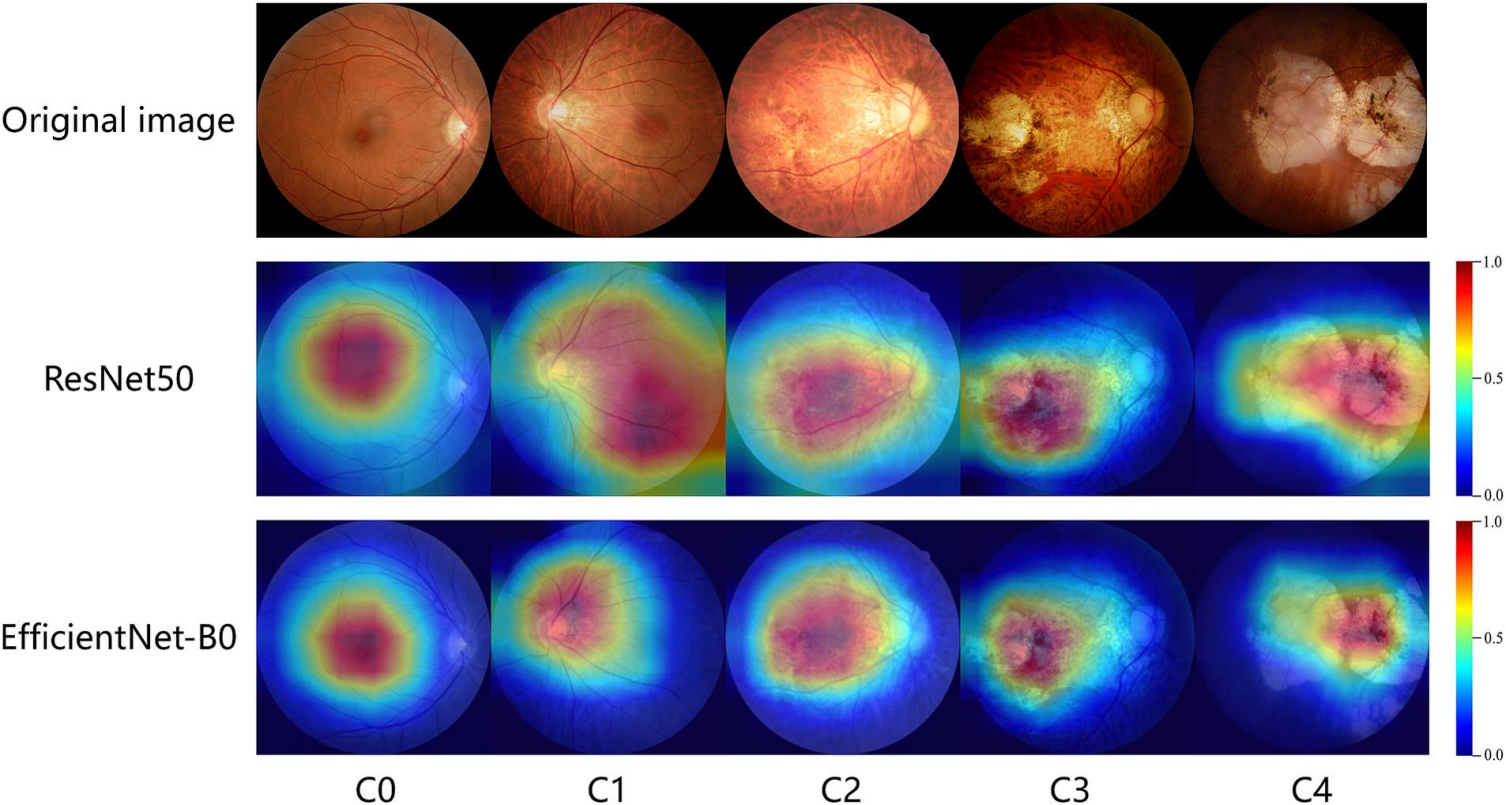
GradCam visualizations of ResNet50 and EfficientNet-B0 diagnoses for myopic maculopathy levels of C0-C4. The redder the area in the heatmap, the more it contributes to the model’s decision-making.

Grad-CAM works by calculating feature maps from convolutional layers and highlighting the areas the model focuses on through weighted summation, which means it only applies to convolutional neural network (CNN) models. In this study, ResNet50 and EfficientNet-B0 are the CNN models used. We input original images of different types of MM into these models and use the Grad-CAM++ algorithm to generate heatmaps. The heatmaps highlight the areas most important for classification with bright colors like red and yellow. The study shows that the heatmaps effectively highlight lesion areas in the fundus images, such as retinal vessels, choroidal atrophy, and the macula. Figure 6 shows representative examples of heatmaps for MM levels C0-C4.

## 4 Discussion

Based on fundus images, this study developed artificial intelligence models to identify no myopic retinopathy, tessellated fundus, diffuse chorioretinal atrophy, patchy chorioretinal atrophy, and macular atrophy. The outputs of these models are fused using the weighted voting method from ensemble learning. Subsequently, all models and their ensemble results are evaluated. Our findings reveal that after ensembling, the models outperform all individual deep learning models (ResNet50, EfficientNet-B0, ViT, CLIP, and RETFound), demonstrating robustness and generalization ability.

The advantage of this work lies in the adoption of multiple advanced classification models. For instance, ResNet50, a widely used and well-performing CNN model, is extensively applied in various visual tasks and demonstrates high accuracy and stability. EfficientNet is a lightweight model that enhances performance through compound scaling methods without increasing model complexity, making it particularly suitable for resource-constrained environments. ViT is a model based on the self-attention mechanism that challenges the dominance of traditional CNNs in visual tasks, effectively capturing global features in images and showing exceptional performance, especially when handling large-scale datasets. CLIP is a multimodal model capable of processing both image and text data. Trained on a large-scale image-text paired dataset, it exhibits strong cross-modal transfer learning capabilities and can be applied to various downstream visual tasks. RETFound is a recently proposed model trained using self-supervised learning on 1.6 million unlabeled retinal images, then adapted to disease monitoring tasks with specific labels, making it particularly suitable for medical image analysis. Finally, this work innovatively employs a voting method in ensemble algorithms, integrating these different types of models, thereby enhancing the robustness and generalization of classification by ensuring diversity and complementarity among models.

During the experiments, we observed that the model performed best on the training set, with performance on the validation set being similar to that on the test set. This consistency indicates that the model has a strong understanding of the features needed to classify myopic maculopathy. It means that the model can generalize to new data, which is important for clinical applications. Additionally, five-fold cross-validation helps ensure stable performance across different data splits.

In analyzing the misclassified images, we noticed that many shared visual similarities with the incorrect categories. This overlap makes it hard for the model to distinguish between certain lesions. To tackle this, future research will focus on strategies like contrastive learning to improve classification of these challenging samples. Additionally, using hard sample mining could help the model better differentiate between similar categories, leading to improved accuracy in clinical applications.

Grad-CAM was applied to visualize the decision-making process. This technique helps identify the areas of the input image that the model focuses on when making predictions. This provides interpretability by revealing how models make decisions, helping clinicians understand and trust AI predictions, which is crucial for clinical adoption.

This study has some limitations. First, in the dataset used for the research, fundus images labeled as C0 (no myopic retinopathy) were defined as free of any lesions. However, in a real clinical setting, some eyes may not have high myopia-related maculopathy but may still have other types of retinal diseases. Similarly, for images of other disease levels, patients in the sample only had a specific single disease, meaning the model did not encounter cases with multiple coexisting retinal diseases during training. This may lead to inaccurate classifications in practical applications. Secondly, our dataset is sourced from a single device, performance may decline when validating images from different fundus cameras due to variations in imaging protocols and quality, which can affect the model’s ability to generalize. Additionally, although quality control measures were implemented in the study to exclude low-quality images, such image quality issues remain common in real-world scenarios. Finally, the application of automatic image quality assessment techniques (31, 32) is crucial, as they can help identify substandard images and alert operators. Finally, while the ensemble method improved diagnostic performance, integrating multiple models also reduced efficiency (33).

Future improvements could include the following: (1) expanding the dataset to enhance its diversity and improve the model’s generalization ability by acquiring fundus photographs from different imaging devices and including cases with various coexisting retinal diseases. Additionally, incorporating external validation is crucial to ensure that the model performs reliably across diverse clinical settings; (2) incorporating automatic image quality assessment techniques to automatically exclude substandard images; (3) optimizing the ensemble model’s algorithm by removing models that do not significantly contribute to the ensemble results or replacing them with more efficient models.

## 5 Conclusion

In conclusion, our study successfully developed an artificial intelligence model capable of automatically identifying no myopic retinopathy, tessellated fundus, diffuse chorioretinal atrophy, patchy chorioretinal atrophy, and macular atrophy from fundus images. By utilizing various advanced deep learning models, including ResNet50, EfficientNet-B0, ViT, CLIP, and RETFound, and innovatively employing a weighted voting ensemble algorithm, we significantly enhanced the model’s classification accuracy and robustness. The results demonstrate that the ensemble model outperforms individual models across multiple metrics, particularly exhibiting strong robustness and generalization ability in the analysis of complex fundus images. This system has the potential to assist ophthalmologists in accurately and promptly identifying the causes of myopic macular lesions, thereby improving patient visual outcomes by enabling targeted treatment at an early stage.

## Data Availability

The datasets presented in this article are not readily available because the data is not public for ethical reasons. Research related requests to access the datasets should be directed to the corresponding author.
